# The effects of familial factors on the early childhood caries of preschool children: a cross-sectional study

**DOI:** 10.1186/s12903-025-06140-w

**Published:** 2025-06-05

**Authors:** Zeynep Aslı Güçlü, Cansu Bilge Karadağ

**Affiliations:** Department of Pediatric Dentistry, Faculty of Dentistry, Erciyes University, Kayseri, 38039 Türkiye

**Keywords:** Child, Dental caries, Indicators, Oral hygiene, Oral hygiene index, Public health

## Abstract

**Background:**

Early childhood caries (ECC) are highly prevalent globally. This study aims to identify evidence-based familial factors to increase the risk-based management of ECC.

**Methods:**

A total of 405 children (aged 0–72 months) presented to the pediatric dentistry department within 2 years were observed via a dental mirror and explorer under dental unit lighting. ECC was diagnosed via visual examination using World Health Organization (WHO) and International Caries Detection and Assessment System (ICDAS) scores. A total of 405 mothers and 399 fathers were interviewed in this study, and a questionnaire regarding parental variables was completed.

**Results:**

Most parents with low education levels visit the dental clinic when their child experiences pain or any complaint within 0–72 months. Maternal age was found to affect DMFT (Decayed, Missed, and Filled Permanent Teeth) scores. A statistically significant difference was found between the mean DMFT score according to the age of the mother at the time of delivery (*p* = 0.045). This study revealed a greater tendency of parents with a high education status to use fluoride. Parents of children without ECC were significantly more likely to have good oral hygiene habits, especially those with dental floss.

**Conclusion:**

ECC was found to be related to parents’ habits and attention. Health providers should consider parents’ knowledge, be aware of many aspects that could contribute to caries balance, and provide anticipatory guidance to parents regarding appropriate dental visits.

**Supplementary Information:**

The online version contains supplementary material available at 10.1186/s12903-025-06140-w.

## Introduction

Despite all the advancements in preventative dentistry, ECC is one of the most significant health issues in preschool-aged children in both industrialized and developing countries [1]. It is a disorder that affects children who are 71 months old or younger and have one or more deciduous teeth with caries (with or without cavitation), tooth loss due to caries, or a filled tooth [2]. 

Many risk factors characterize ECC. ECC is highly linked to frequent sugar consumption, particularly before bed [3, 4]. Inadequate oral hygiene, characterized by infrequent teeth brushing and visible plaque, elevates the risk of ECC [5, 6]. Socioeconomic determinants, including parental education and occupation, influence outcomes [7, 8]. Maternal variables, such as untreated dental caries and tobacco use, substantially affect the risk of early childhood caries [9]. Additional risk factors include salivary pH [8], lack of a dental home [5], and prolonged breastfeeding or bottle-feeding at night [4]. Furthermore, ECC development is influenced by the nature of the oral microbiome, namely elevated levels of *Lactobacilli* and *Streptococci* [6]. It is essential to comprehend these risk variables to create successful prevention plans. The development of caries is strongly influenced by the child’s oral hygiene practices, fluoride intake, and brushing engagement. Dental caries can be avoided by removing dental plaque with equipment such as toothbrushes and dental floss [4, 5, 6, 7, 8]. 

Addressing risk indicators and their correlation with ECC is necessary to facilitate the development of preventive measures and health care programs [10]. Early childhood development requires parental care, and through their knowledge and behaviors, parents actively influence their children’s habits throughout their lives. The family’s or caregiver’s degree of education, physical and mental health, opportunities, and financial resources influence the child’s habits [11, 12]. Therefore, among the many factors contributing to the development of ECC, which has a complex etiology, family factors need to be studied further [10, 13]. 

The objective of this hospital-based cross-sectional study was to assess how parental education, knowledge, and oral hygiene practices affect the dental health of children. The null hypothesis was that parents’ education, behaviors, and habits do not affect ECC or its severity.

## Methods

A total of 405 patients aged 71 months and younger, 191 boys and 214 girls, underwent dental examinations at the University Faculty of Dentistry, Pediatric Dentistry Department, between 2021 and 2022. The risk assessment questionnaire concerning their habits was completed by the parents, who applied to the clinic in a face-to-face question-and-answer format. A total of 405 mothers and 399 fathers answered the questionnaire, and 6 fathers were not included because of their parent’s death or divorce. The study was carried out in city x, which is located in the middle of region x. The survey, given as supplementary files, was based on a comprehensive literature review [11-32]. 

When analyzing the connection between ECC and familial influences,

- Age of the mother at the time of birth.
- Complications experienced by the mother during pregnancy.
- Maternal smoking and drug use during pregnancy.
- Education levels of mothers and fathers (primary school and before, secondary school, high school, associate degree, bachelor’s degree, master’s degree, doctorate).
- Parents’ employment status.
- Parents’ tooth brushing habits, brushing frequency, toothpaste preferences, and flossing status.
- Frequency of dental visits by parents.
- Oral care education was evaluated.

To assess parents’ knowledge and attitudes depending on their education levels and dental visit frequency,

- Relationships between parents’ frequency of dental visits.
- The relationship between parental preference for toothpaste.
- Relationships among the kinds of toothpaste used by children.
- Relationship between using the same spoon with their children.
- Relationship between the child’s ability to chew food.
- The answers to the question “Is it necessary to treat deciduous teeth?”
- The relationship between the answers to the question “Do you think your child’s oral care is adequate?” was determined.

Parents who refused to participate (*n* = 10) were excluded from the study. Fifteen children attempted to undergo assessment but were omitted from the study due to inadequate cooperation. The parents of the patients included in the study were asked to sign an informed consent form before the examination. Ethical approval for the study was obtained from the Erciyes University Clinical Research Ethics Committee and was carried out under the Principles of the Declaration of Helsinki. (Decision No: 2021/504).

Intraoral examinations of the patients were performed by the same pediatric dentist in the dental unit under bright light using a mirror, probe, and air-water spray. The detected caries lesions were recorded in the regions allocated for the DMFT index and ICDAS scoring on the risk assessment form. ICDAS and DMFT data were collected separately by clinical observation.

The examiner is trained on examination criteria under the leadership of pediatric dentistry academic professionals. The training consists of evaluating intraoral digital images and caries ratings, followed by one month of specialized clinical training. In the subsequent month, two independent examiners evaluated the examiner on 75 cases, and a success rate of 98% in terms of validity and reliability was accepted before trial. This high accuracy rate indicates the observer’s assessments were highly consistent with independent experts’ evaluations of individual teeth concerning the ICDAS system and DMFT score.

For the region allocated to the DMFT index, teeth were recorded as untreated decayed teeth (d), extracted teeth due to caries (m), and filled teeth (f). Initial caries lesions where no probe was attached were excluded. Nonerupted teeth were not included. For ICDAS scoring, 0 indicates no change in the enamel surface after drying the tooth surface for 5 s, 1 indicates visible opacity and discoloration on the enamel surface at the fissure entrance after 5 s of air, and 1 indicates a significant change visible on the moist tooth surface; 2 indicates the presence of lesions visible when the tooth was dried, 3 indicates localized fractures on the enamel surface, 4 indicates yellow‒brown reflection on the dentine, 5 indicates visible cavitation involving the dentine, and 6 indicates extensive cavitation on the dentine covering more than half of the occlusal surface. After scoring each tooth individually, the patient was evaluated according to the highest score obtained by the patient.

### Statistical analysis

The data were analyzed with IBM SPSS V23. Compliance with a normal distribution was examined by the Kolmogorov‒Smirnov and Shapiro‒Wilk tests. The chi-square test was used to compare categorical variables by group. The Mann‒Whitney U test was used to compare nonnormally distributed data according to paired groups. One-way analysis of variance was used to compare normally distributed data according to groups of three or more variables. Comparisons of nonnormally distributed data according to groups of three or more variables were performed via the Kruskal‒Wallis test. The analysis results are presented as the means ± s. deviations and medians ( minimum-maximum) for quantitative data and frequencies (percentages) for categorical data. The significance level was set at *p* < 0.050.

The data were analyzed with the R program. The effect of independent variables on DMFT and ICDAS values, which do not show normal distribution, was examined by Robust regression analysis using the MASS package. The severity level was taken as *p* < 0.050. In the study, whether the dependent variables, ICDAS and DMFT scores, showed a significant difference according to different categorical variables was primarily analyzed using comparison tests. As a result of these comparisons, the variables that showed a statistically significant difference were included as independent variables in the multiple regression models. The selection of independent variables in the regression models was based on these preliminary analyses. Multicollinearity among independent variables was assessed using Variance Inflation Factor (VIF), which indicated no problems. As the dependent variables did not follow a normal distribution, robust regression analysis was employed to ensure valid results. VIF values were reported to confirm the model’s reliability and validity.

The sample width was calculated via the G*Power V. 3.1.9.6 program. In the study with 95% confidence (1-α) and 95% test power (1-β), f = 0.197 effect size, the total number of cases required was 402 [30]. 

## Results

The study evaluated 405 children, including 191 boys (47.2%) and 214 girls (52.8%), who applied for examination between 2021 and 2022. The mean age of the children was 56.75 ± 11.24 months. The mean DMFT and ICDAS scores of the children included in the study were 8.79 ± 4.33 and 5.06 ± 1.46, respectively (Table 1). Only 18 children were caries-free, and 387 (95%) had caries.

**Table 1:** Findings related to children

	Frequency (*n*)/Mean ± std. deviation	Percentage (%)/median (min. - max.)
Age		
13-24 Months(1 Years old)	4	1
25-36 Months(2 Years old)	17	4. 2
37-48 Months(3 Years old)	73	18
49-60 Months(4 Years old)	124	30. 6
61-72 Months(5 Years old)	187	46. 2
Age (month)	56. 75 ± 11. 24	59. 00 (13. 00 - 71. 00)
**Gender**		
Boy	191	47. 2
Girl	214	52. 8
DMFT	8. 79 ± 4. 33	9. 00 (0. 00 - 20. 00)
ICDAS	5. 06 ± 1. 46	5. 00 (0. 00 - 6. 00)
**HAS ECC**		
Yes	387	95. 6
No	18	4. 4
	**Frequency (n)**	**Percentage (%)**
**Dental Cleaning After Night Feeding**		
Yes	149	36. 8
No	256	63. 2
**Age of Starting Brushing**		
6- 12 Months	13	3. 2
After 1 year of age	171	42. 2
After 3 years of age	172	42. 5
Never	49	12. 1
**Daily Brushing Frequency**		
Never	49	12. 13
1 Time	300	74. 07
2 or More	56	13. 8
**When is Brushing?**		
Morning	74	20. 8
Evening	140	39. 3
Changes	86	24. 1
Morning and Evening	56	15. 7
**Does he/she use toothpaste for every brushing?**		
Yes	331	93
No	25	7
**Does Toothpaste Contain Fluorine?**		
Yes	67	18. 8
No	112	31. 7
I do not know	177	49. 5
**How is brushing done?**		
By Parent	67	18. 8
By Child under Parental Supervision	208	58. 4
Child Alone	81	22. 8

The factors related to mothers are given in Table 2, and fathers are in Table 3.

**Table 2:** Frequency distribution of factors related to the mother

	FREQUENCY (*n*)	Percentage (%)
Age of the mother at the time of birth		
18-24	106	26,2
25-29	167	41,2
30 and Above	132	32,6
**Mother’s Education Level**		
Primary School and Less	63	15,6
Middle School	96	23,7
High School	118	29,1
Associate Degree	51	12,6
Bachelor’s degree	65	16
Master’s Degree	9	2,2
PhD	3	0,7
**Does the mother work?**		
Yes	73	18
No	332	82
**Does the Mother Brush Her Teeth**		
Yes	385	95,1
No	20	4,9
**Mother’s Brushing Frequency**		
Occasionally	44	11,4
Once a day	212	55,06
2 or More Per Day	129	33,54
**Does the Mother Use Toothpaste**		
Yes	382	99,2
No	3	0,8
**Does the Mother Use Dental Floss**		
Yes	84	20,7
No	321	79,3

**Table 3:** Frequency distribution of father-related factors

	FREQUENCY (*n*)	Percentage (%)
Father’s Education Level		
Primary School and Before	53	13,3
Middle School	68	17
High School	141	35,3
Associate Degree	33	8,3
Bachelor’s degree	81	20,3
Master’s Degree	17	4,3
PhD	6	1,5
**Does the Father Brush His Teeth**		
Yes	351	88
No	48	12
**Father’s Brushing Frequency**		
Occasionally	85	24,2
Once a day	175	49,8
2 or More Per Day	91	26
**Does the Father Use Toothpaste**		
Yes	339	96,4
No	12	3,6
**Does the Father Use Dental Floss**		
Yes	82	20,6
No	317	79,4

The mean DMFT score of mothers who gave birth between 18 and 24 (9.64 ± 4.01) was significantly greater than that of mothers who gave birth between 25 and 29 years of age (8.20 ± 4.47). There was no statistically significant difference in the mean ICDAS score according to the mother’s age at the time of delivery (*p* = 0.082) (Table 4). Tables 4 and 5 show parent-related factors.

**Table 4:** Comparison of DMFT and ICDAS scores according to maternal factors

	DMFT			ICDAS		
	Mean ± Std. deviation	Median (min. - max.)		Mean ± Std. deviation	Median (min. - max.)	
Mother’s Education Level						
Primary school and before	9,95 ± 3,62a	10,00 (2,00 - 20,00)		5,56 ± 0,64	6,00 (4,00 - 6,00)b	
Middle School	9,67 ± 3,31a	10,00 (2,00 - 19,00)		5,50 ± 0,77	6,00 (2,00 - 6,00)b	
High School	8,78 ± 4,50ab	9,00 (0,00 - 20,00)		5,03 ± 1,46	5,00 (0,00 - 6,00)ab	
Associate degree	8,41 ± 4,61ab	8,00 (0,00 - 20,00)		4,86 ± 1,55	5,00 (0,00 - 6,00)ab	
Bachelor’s degree and above	7,01 ± 4,98b	7,00 (0,00 - 20,00)		4,27 ± 2,06	5,00 (0,00 - 6,00)a	
**Test stat.**	5,321			31,635		
p**	**<0**,**001**			**<0**,**001**		
**Does the mother work?**						
Yes	6,45 ± 4,71		6,00 (0,00 - 16,00)	4,18 ± 2,15		5,00 (0,00 - 6,00)
No	9,30 ± 4,08		9,00 (0,00 - 20,00)	5,25 ± 1,17		6,00 (0,00 - 6,00)
**Test stat.**	16285,000			15735,500		
p*	**<0**,**001**			**<0**,**001**		
**Does the Mother Use Dental Floss?**						
Yes	7,51 ± 4,56		8,00 (0,00 - 20,00)	4,57 ± 1,66		5,00 (0,00 - 6,00)
No	9,12 ± 4,22		9,00 (0,00 - 20,00)	5,19 ± 1,37		6,00 (0,00 - 6,00)
**Test stat.**	16342,500			17447,000		
p*	**0**,**003**			**<0**,**001**		

*Mann‒Whitney U test, **One-way analysis of variance, a--b: There is no difference between groups with the same letter, ---: Not compared due to insufficient observations

**Table 5:** Comparison of DMFT and ICDAS scores according to father-related factors

	DMFT		ICDAS	
	Mean ± Std. deviation	Median (min. - max.)	Mean ± Std. deviation	Median (min. - max.)
Father’s Education Level				
Primary school and less	10,34 ± 3,18	10,00 (6,00 - 20,00)a	5,57 ± 0,69	6,00 (3,00 - 6,00)a
Middle School	9,46 ± 4,23	9,00 (0,00 - 20,00)a	5,46 ± 0,95	6,00 (0,00 - 6,00)a
High School	8,99 ± 4,19	9,00 (0,00 - 20,00)a	5,27 ± 1,16	6,00 (0,00 - 6,00)a
Associate degree	9,33 ± 4,48	10,00 (2,00 - 18,00)ab	5,12 ± 1,14	5,00 (1,00 - 6,00)ab
Bachelor’s degree and above	6,91 ± 4,55	7,00 (0,00 - 16,00)b	4,19 ± 2,04	5,00 (0,00 - 6,00)b
**Test stat.**	23,296		42,165	
p**	**<0**,**001**		**<0**,**001**	
**Does the Father Brush His Teeth**				
Yes	8,52 ± 4,34	8,00 (0,00 - 20,00)	4,97 ± 1,51	5,00 (0,00 - 6,00)
No	10,29 ± 4,04	11,00 (0,00 - 19,00)	5,58 ± 0,96	6,00 (0,00 - 6,00)
**Test stat.**	10507,000		10846,000	
p*	**0**,**005**		**<0**,**001**	
**Father’s Brushing Frequency**				
Occasionally	9,41 ± 4,19	9,00 (0,00 - 20,00)	5,19 ± 1,21	5,00 (0,00 - 6,00)b
1 time a day	8,73 ± 4,30	9,00 (0,00 - 20,00)	5,04 ± 1,52	5,00 (0,00 - 6,00)b
2 or more per day	7,74 ± 4,67	8,00 (0,00 - 20,00)	4,66 ± 1,70	5,00 (0,00 - 6,00)a
**Test stat.**	5,659		7,216	
p**	0,059		**0**,**027**	
**Does the Father Use Dental Floss**	Does the Father Use Dental Floss			
Yes	Yes	8,00 (0,00 - 19,00)	4,52 ± 1,77	5,00 (0,00 - 6,00)
No	No	9,00 (0,00 - 20,00)	5,18 ± 1,35	6,00 (0,00 - 6,00)
**Test stat.**	Test stat.		16297,000	
p*	**0**,**009**		**<0**,**001**	

*Mann‒Whitney U test, **Kruskal‒Wallis test, a-b: There is no difference between groups with the same letter

A difference was found in the distribution of toothpaste preferences according to the educational status of the mothers (*p* < 0.001). While the number of mothers who used fluoride was significantly greater in the group with undergraduate and higher education levels, the number of mothers who answered “do not know” was the lowest in the group with undergraduate and higher education levels. Fluoride toothpaste was preferred by 12.7% of those with primary school education, 25% of those with secondary school education, 19.5% of those with high school education, 29.4% of those with an associate degree, and 46.8% of those with a bachelor’s degree or higher (Table 6).

**Table 6:** Comparison results according to mothers’ education level

	Primary school and belove	Middle School	High School	Associate degree	Bachelor’s degree and above	Test stat.	*p*
Brushing Frequency of Children							
Never	9 (14,3)	17 (17,7)	19 (16,1)	4 (7,8)	5 (6,5)	8,75	0,36
Once	47 (74,6)	65 (67,7)	84 (71,2)	37 (72,5)	62 (80,5)		
2 or more	7 (11,1)	14 (14,6)	15 (12,7)	10 (19,6)	10 (13)		
**Frequency of Parental Visits to the Dentist**							
When I experience pain or any kind of distress	49 (77,8)	80 (83,3)	101 (85,6)	39 (76,5)	53 (68,8)	11,48	0,18
Once a year	13 (20,6)	12 (12,5)	14 (11,9)	9 (17,6)	20 (26)		
Every 6 months	1 (1,6)	4 (4,2)	3 (2,5)	3 (5,9)	4 (5,2)		
**Parents’ Choice of Toothpaste**							
Fluoride	8 (12,7)a	24 (25)a	23 (19,5)a	15 (29,4)ab	36 (46,8)b	37,59	**<0**,**01**
Fluoride Free	8 (12,7)	10 (10,4)	19 (16,1)	9 (17,6)	17 (22,1)		
I do not know	47 (74,6)a	62 (64,6)a	76 (64,4)a	27 (52,9)ab	24 (31,2)b		
**Do You Use the Same Spoon with Your Child?**							
Yes	6 (9,5)	16 (16,7)	14 (11,9)	4 (7,8)	14 (18,2)	4,83	0,31
No	57 (90,5)	80 (83,3)	104 (88,1)	47 (92,2)	63 (81,8)		
**Do you chew your child’s food?**							
Yes	2 (3,2)	5 (5,2)	9 (7,7)	1 (2)	2 (2,6)	4,33	0,36
No	61 (96,8)	91 (94,8)	108 (92,3)	50 (98)	75 (97,4)		
**Do you think it is necessary to treat deciduous teeth?**							
Yes	32 (50,8)	61 (63,5)	72 (61)	33 (64,7)	61 (79,2)	15,34	0,05
No	6 (9,5)	5 (5,2)	6 (5,1)	5 (9,8)	3 (3,9)		
No opinion	25 (39,7)	30 (31,3)	40 (33,9)	13 (25,5)	13 (16,9)		
**Do you think your child’s oral care is adequate?**							
Yes	5 (7,9)	12 (12,5)	16 (13,6)	11 (21,6)	14 (18,2)	15,38	0,05
No	42 (66,7)	60 (62,5)	70 (59,3)	35 (68,6)	54 (70,1)		
I do not know	16 (25,4)	24 (25)	32 (27,1)	5 (9,8)	9 (11,7)		

*Chi-square test, a-b: No difference between groups with the same letter, frequency (percentage)

A statistically significant difference was found between the distributions of toothpaste preferences according to the father’s education level (*p* < 0.001). While the rate of using fluoride toothpaste by fathers with undergraduate and higher education levels was significantly higher than that of the other fathers, the rate of not knowing the fluoride content was lower than that of the other fathers. Fluoride toothpaste was preferred by 13.2% of those with primary school education, 17.6% of those with secondary school education, 24.8% of those with high school education, 30.3% of those with an associate degree, and 39.4% of those with a bachelor’s degree or higher. No statistically significant difference was found between the distributions of other variables according to the father’s educational status (*p* > 0.050) (Table 7).

**Table 7:** Comparison results according to fathers’ education level

	Primary school and belove	Middle School	High School	Associate degree	Bachelor’s degree and above	Test stat.	*p*
Brushing Frequency of Children							
Never	11 (20,8)	11 (16,2)	21 (14,9)	2 (6,1)	8 (7,7)	11,63	0,17
Once	33 (62,3)	44 (64,7)	103 (73)	26 (78,8)	84 (80,8)		
2 or more	9 (17)	13 (19,1)	17 (12,1)	5 (15,2)	12 (11,5)		
**Frequency of Parental Visits to the Dentist**							
When I experience pain or any kind of distress	43 (81,1)	56 (82,4)	114 (80,9)	25 (75,8)	80 (76,9)	4,33	0,83
Once a year	9 (17)	9 (13,2)	22 (15,6)	8 (24,2)	19 (18,3)		
Every 6 months	1 (1,9)	3 (4,4)	5 (3,5)	0 (0)	5 (4,8)		
**Parents’ Choice of Toothpaste**							
Fluoride	7 (13,2)a	12 (17,6)a	35 (24,8)ab	10 (30,3)ab	41 (39,4)b	28,51	**<0**,**01**
Fluoride Free	6 (11,3)	8 (11,8)	21 (14,9)	4 (12,1)	23 (22,1)		
I do not know	40 (75,5)a	48 (70,6)a	85 (60,3)a	19 (57,6)ab	40 (38,5)b		
**Do You Use the Same Spoon with Your Child?**							
Yes	8 (15,1)	13 (19,1)	20 (14,2)	0 (0)	11 (10,6)	8,08	0,09
No	45 (84,9)	55 (80,9)	121 (85,8)	33 (100)	93 (89,4)		
**Do you chew your child’s food?**							
Yes	2 (3,8)	2 (2,9)	9 (6,4)	1 (3)	4 (3,8)	1,92	0,75
No	51 (96,2)	66 (97,1)	131 (93,6)	32 (97)	100 (96,2)		
**Do you think it is necessary to treat deciduous teeth?**							
Yes	33 (62,3)	40 (58,8)	78 (55,3)	26 (78,8)	76 (73,1)	12,85	0,12
No	4 (7,5)	4 (5,9)	10 (7,1)	1 (3)	6 (5,8)		
No opinion	16 (30,2)	24 (35,3)	53 (37,6)	6 (18,2)	22 (21,2)		
**Do you think your child’s oral care is adequate?**							
Yes	6 (11,3)	8 (11,8)	14 (9,9)	6 (18,2)	23 (22,1)	12,08	0,15
No	38 (71,7)	44 (64,7)	90 (63,8)	19 (57,6)	65 (62,5)		
I do not know	9 (17)	16 (23,5)	37 (26,2)	8 (24,2)	16 (15,4)		

*Chi-square test, a-b: No difference between groups with the same letter, frequency (percentage)

The regression model established to examine the effect of independent variables on DMFT values was statistically significant (Table 8) (F = 4.91, *p* < 0.01). DMFT values of non-working mothers were obtained 2.37 units higher than those who worked (*p* < 0.01). DMFT values of undergraduates and above were 1.83 units less than those in primary school and before (*p* = 0.03). DMFT values of fathers who did not brush their teeth were 1.24 units higher than those who brushed (*p* = 0.04). Other variables did not have a statistically significant effect on DMFT values (*p* > 0.05). Together with the independent variables in the model, the dependent variable is explained at a rate of 15.2%.

**Table 8:** Investigation of the effect of independent variables on DMFT by robust regression analysis

	β1 (95% CI)	Q. Error	β2	t	*p*	VIF
Constant	7 (4,83 - 9,16)	1,10	0,00	6,36	**<0.01**	---
Mother’s age at the time of birth (ref: 18-24)						
25-29	-0,76 (-1,75 - 0,23)	0,50	-0,09	-1,51	0,13	1,69
30 and above	-0,49 (-1,6 - 0,63)	0,57	-0,06	-0,86	0,39	1,95
Mother’s Educational Status (Reference: Primary school and before)						
Secondary school	0,28 (-1,13 - 1,68)	0,72	0,03	0,38	0,70	2,64
High school	-0,57 (-1,95 - 0,81)	0,70	-0,06	-0,81	0,42	2,73
Associate Degree	-0,3 (-1,96 - 1,37)	0,85	-0,02	-0,35	0,73	2,20
Bachelor’s degree and above	0,27 (-1,53 - 2,07)	0,92	0,03	0,29	0,77	3,55
Does the mother work (reference: yes)	2,37 (1,15 - 3,59)	0,62	0,22	3,83	**<0.01**	1,54
Does Mom Floss (Reference: Yes)	0,67 (-0,38 - 1,73)	0,54	0,07	1,25	0,21	1,29
Father’s Educational Status (Reference: Primary school and before)						
Secondary school	-0,91 (-2,4 - 0,58)	0,76	-0,08	-1,20	0,23	2,24
High school	-0,97 (-2,28 - 0,34)	0,67	-0,11	-1,46	0,15	2,78
Associate Degree	-0,21 (-2,04 - 1,61)	0,93	-0,01	-0,23	0,82	1,81
Bachelor’s degree and above	-1,83 (-3,44 - -0,22)	0,82	-0,20	-2,23	**0**,**03**	3,56
Does dad brush his teeth (reference: yes)	1,24 (0,06 - 2,43)	0,60	0,10	2,06	**0**,**04**	1,07
Does Dad Floss (Reference: Yes)	0,69 (-0,36 - 1,74)	0,53	0,07	1,29	0,20	1,28

F=4.91, *p*<0.01, R2=0.152, β1 (95% CI): Non-standardized beta coefficient (95% confidence interval), β2: Standardized beta coefficient

The regression model established to examine the effect of independent variables on DMFT values was statistically significant (F=4.91, *p*<0.01). DMFT values of non-working mothers were obtained 2.37 units higher than those who worked (*p*<0.01). DMFT values of undergraduate and above were 1.83 units less than those in primary school and before (*p*=0.03). DMFT values of fathers who did not brush their teeth were 1.24 units higher than those who brushed (*p*=0.04). Other variables did not have a statistically significant effect on DMFT values (*p*>0.05). Together with the independent variables in the model, the dependent variable is explained at a rate of 15.2%

The regression model established to examine the effect of independent variables on ICDAS values was statistically significant (Table 9) (F = 5.05, *p* < 0.01). ICDAS values of non-working mothers were obtained 0.32 units higher than those who worked (*p* = 0.02). The ICDAS values of mothers who did not use dental floss were 0.33 units higher than those who used dental floss (*p* = 0.01). ICDAS values of undergraduate and above were 0.5 units less than those in primary school and before (*p* = 0.01). There was no statistically significant effect of other variables on ICDAS values (*p* > 0.050). Together with the independent variables in the model, the dependent variable is explained at a rate of 15.6%.

**Table 9:** Investigation of the effect of independent variables on DMFT by robust regression analysis

	β1 (95% CI)	Q. Error	β2	t	*p*	VIF
Constant	7 (4,83 - 9,16)	1,10	0,00	6,36	**<0.01**	---
Mother’s age at the time of birth (ref: 18-24)						
25-29	-0,76 (-1,75 - 0,23)	0,50	-0,09	-1,51	0,13	1,69
30 and above	-0,49 (-1,6 - 0,63)	0,57	-0,06	-0,86	0,39	1,95
Mother’s Educational Status (Reference: Primary school and before)						
Secondary school	0,28 (-1,13 - 1,68)	0,72	0,03	0,38	0,70	2,64
High school	-0,57 (-1,95 - 0,81)	0,70	-0,06	-0,81	0,42	2,73
Associate Degree	-0,3 (-1,96 - 1,37)	0,85	-0,02	-0,35	0,73	2,20
Bachelor’s degree and above	0,27 (-1,53 - 2,07)	0,92	0,03	0,29	0,77	3,55
Does the mother work (reference: yes)	2,37 (1,15 - 3,59)	0,62	0,22	3,83	**<0.01**	1,54
Does Mom Floss (Reference: Yes)	0,67 (-0,38 - 1,73)	0,54	0,07	1,25	0,21	1,29
Father’s Educational Status (Reference: Primary school and before)						
Secondary school	-0,91 (-2,4 - 0,58)	0,76	-0,08	-1,20	0,23	2,24
High school	-0,97 (-2,28 - 0,34)	0,67	-0,11	-1,46	0,15	2,78
Associate Degree	-0,21 (-2,04 - 1,61)	0,93	-0,01	-0,23	0,82	1,81
Bachelor’s degree and above	-1,83 (-3,44 - -0,22)	0,82	-0,20	-2,23	**0**,**03**	3,56
Does dad brush his teeth (reference: yes)	1,24 (0,06 - 2,43)	0,60	0,10	2,06	**0**,**04**	1,07
Does Dad Floss (Reference: Yes)	0,69 (-0,36 - 1,74)	0,53	0,07	1,29	0,20	1,28

F=4.91, *p*<0.01, R2=0.152, β1 (95% CI): Non-standardized beta coefficient (95% confidence interval), β2: Standardized beta coefficient

The regression model established to examine the effect of independent variables on DMFT values was statistically significant (F=4.91, *p*<0.01). DMFT values of non-working mothers were obtained 2.37 units higher than those who worked (*p*<0.01). DMFT values of undergraduate and above were 1.83 units less than those in primary school and before (*p*=0.03). DMFT values of fathers who did not brush their teeth were 1.24 units higher than those who brushed (*p*=0.04). Other variables did not have a statistically significant effect on DMFT values (*p*>0.05). Together with the independent variables in the model, the dependent variable is explained at a rate of 15.2%

## Discussion

In our study, the DMFT and ICDAS indices were used. Although the DMFT index is preferred due to its ease of application and high repeatability, it has been supplemented by the ICDAS index because it does not fully reveal the severity of carious lesions and cannot assess initial carious lesions [33]. According to this system, 84.7% of children had at least one advanced carious lesion (ICDAS 5–6) in their primary teeth. This situation indicates that, although the number of children participating in the study with cavities, filled, or extracted teeth may not be high, most of them have deep dentin caries, revealing that the DMFT rates reflect a more severe issue than initially apparent. Including non-cavitated lesions in ICDAS significantly impacts caries prevalence and extent estimates compared to standard WHO criteria [34]. 

In this study, maximum ICDAS, a summary measure of caries that reflects the severity of lesions, was used. In clinical practice, the most severe lesion often determines the urgency and type of treatment. Maximum ICDAS addresses this clinical need and might be relevant for treatment decisions based on the most severe lesion. Since deep dentin caries can lead to pain and infection, approaches should be adopted not only to reduce the prevalence of caries but also to decrease the severity of the caries [33]. The maximum ICDAS score has also been used in two other studies [35, 36]. On the other hand, the total or mean ICDAS is more suitable for population-level comparisons (42), have shown showing strong correlations with caries lesions at different severity levels [37, 38]. 

Parents are the most important role models for children from the beginning of their lives because they serve as mirrors to them with their knowledge, attitudes, and behaviors. What children observe in their parents shapes their habits [12, 13]. 

Early motherhood is considered a risk factor for dental caries because the woman may be unable to provide adequate care for both herself and her kid [11] Although the mean ICDAS score was greater in this study, the difference was not statistically significant (*p* = 0.082), suggesting that the children of mothers who gave birth before the age of 24 had a mean DMFT that was statistically greater (*p* = 0.045). Hallett et al. [14] reported that the prevalence of ECC was lower in children whose mothers were 24 years of age or younger at birth. Mattila et al. [25] found that children of mothers and fathers younger than 25 years of age had a higher rate of being affected by dental caries, similar to the findings of this study. Children of mothers with at least 13 years of education or more, as well as children of fathers with at least 15 years of education, had lower rates of dental caries, according to Tanaka et al. [28]. Chouchene et al. [21] discovered a statistically significant correlation between parental education levels and ECC. A total of 67.9% of parents of caries-free children attended college. In a study by Boonyawong et al. [20], the incidence rate of dental caries was considerably greater in children whose mothers had completed compulsory education than in children whose mothers had completed higher education. The father’s educational status was not linked to the child’s experience with caries, according to the same study. A poor level of maternal education was strongly linked with ECC in a study by Hallett et al. [14]. Children of mothers who completed their basic school were shown to have a considerably higher mean DMFT in the study by Onur et al. [27]. The risk of caries in the child was 2.6 times greater when the mother’s education level was lower. According to the same study, children whose fathers had earned at least a bachelor’s degree had a much lower rate of DMFT than other children did. According to Narang et al. [26], mothers are less influenced by ECC when they are more educated. According to the study, children of working mothers had much lower rates of dental caries. According to Congiu et al. [22], mothers who worked and had better education levels had children with fewer dental caries. In contrast to this study, Pinto et al. [11] and Upadhyay et al. [29] found no significant difference between maternal employment status and education level or dental caries. Johansson et al. [24] reported that parental education level was not associated with dental caries in children.

In our study, the mean DMFT and ICDAS scores decreased as mothers’ education levels increased. In addition, the educational level of the mothers also affected their preference for the use of fluoride toothpaste. Mothers who graduated from undergraduate and higher programs preferred fluoride toothpaste. The mother’s employment status also significantly affected our study’s results. Children of working mothers had significantly lower mean scores. These results suggest that increased education levels and employment increased mothers’ awareness; accordingly, mothers were more careful about their children’s oral health. In our study, early brushing commencement and twice-daily brushing reduced DMFT rates but did not affect ICDAS rates. Therefore, until proper cleaning is performed by brushing, the severity of caries cannot be minimized.

The rate of fluoride toothpaste preference of these fathers was significantly greater than that of the other fathers. (*p* < 0.001) As the education level increased, the rate of fluoride toothpaste preference among fathers increased.

Mothers are usually the main caregivers in early childhood and are the people who are most in contact with the child. For this reason, they are the primary sculptors for the habits that will develop in the children. The oral hygiene habits and oral health status of the mother affect the oral health of the child [11]. Kocaman et al. [19] reported that the regular brushing habits of mothers decreased the rate of caries in their children. Onur et al. [27] reported that mothers with flossing habits paid more attention to the oral health of their children and that the risk of caries increased 1.89 times in mothers who did not floss.

Mattila et al. [25] showed that parents with irregular brushing habits and not using toothpaste increased the rate of dental caries in their children. Ivančević et al. [23] found that fathers’ low health awareness was associated with ECC. Yiğit et al. [30] found that parents’ tooth brushing habits, dental floss use, and mothers’ mouthwash use were significantly associated with DMFS values in children. In addition, the DMFS values of the children of parents with a gingival and oral hygiene index of 3 were significantly greater than those of the children of parents with a gingival and oral hygiene index of 0. In our study, although the presence of the mother’s toothbrushing habit and the increase in brushing frequency significantly decreased the DMFT and ICDAS values. Flossing was significantly associated with lower ICDAS values.

Children’s caries status was found to be strongly correlated with fathers’ brushing techniques, frequency, and flossing. Children of fathers who brushed their teeth had significantly reduced ECC values.

### Limitations of the study

The study population is limited in that it comprises children and parents who attend one dental clinic. Field surveys and dental observation can yield more accurate findings. The survey was performed by asking face-to-face questions, which could be a limitation for making parents feel uncomfortable with being judged or self-reporting. A cross-sectional design, and lack of dietary information that influences caries development are limitations of this study. This study did not involve caries activity testing, which would be beneficial as there is a strong correlation between dental caries and oral bacteria; lack of microbiological data was a limitation. A single examiner conducted the study, and although it would have been preferable to have two or more examiners, measures have been taken to ensure the validity of the data. The maximum ICDAS score is a limitation, as it may not fully represent the patient’s overall caries condition. Other measures, such as the total ICDAS score or the mean ICDAS score, may be utilized to achieve a more thorough evaluation.

## Conclusion

It can be concluded that parents who are more motivated to maintain good oral hygiene reduce their children’s risk of developing cavities. Additionally, more knowledgeable parents preferred fluoride paste in more significant quantities. Children whose parents flossed regularly had considerably lower DMFT and ICDAS scores. The practice of flossing was considered a sign of awareness of oral hygiene. This study highlights the importance of parental engagement for children’s oral health.

Mothers should receive education during pregnancy, especially early in this society. Improving parental knowledge can be a major advantage for public oral health service resource allocation, as it has a significant role in ECC. Clinicians should thoroughly understand caregivers’ characteristics to manage parent-assisted oral health.

## Electronic supplementary material

Below is the link to the electronic supplementary material.

Supplementary Material 1

Supplementary Material 2

Supplementary Material 3

## Data Availability

The patient record data used in this study anonymized and is available upon request from the corresponding author, subject to ethical approval and a signed Data Use Agreement. The data is managed and stored following the ethical guidelines and data security protocols of Erciyes University Faculty of Dentistry.

## Abbreviations

ECC: Early childhood caries
WHO: World Health Organization
ICDAS: International Caries Detection and Assessment System
DMFT: Decayed, Missed, and Filled Permanent Teeth
