# Supplementary material for: The effects of familial factors on the early childhood caries of preschool children: a cross-sectional study
Source: BMC Oral Health. 2025 Jun 5;25:920. doi: 10.1186/s12903-025-06140-w (PMC12142995; doi:10.1186/s12903-025-06140-w)
Supplement: Supplementary file 1 — Supplementary Material 1 [file 12903_2025_6140_MOESM1_ESM.docx]

**Survey (1-21)**

**CHAPTER 1 – PATIENT INFORMATION**

| **Name Surname:** |  | |
| --- | --- | --- |
| **File No:** |  | |
| **Age:** |  | |
| **Gender:** |  | |
| **The person who brought it to the examination:** |  | |
| **Phone:** |  | |
| **Nationality:** | **TC ( )** | **Refugee ( )** |

**CHAPTER 2 – DENTAL EXAMINATION**

**2.1. DMFT INDEX**

| **t** |  |
| --- | --- |
| **d** |  |
| **m** |  |
| **f** |  |

**D: Untreated decayed tooth**

**M: Extracted tooth due to caries**

**F: Filled tooth**

**2.2. ICDAS**

**0: No change on enamel surface after drying for 5 sec**

**1: Visible opacity and discoloration on the enamel surface at the fissure entrance after drying for 5 seconds**

**2: Visible visible change on the moist tooth surface, visible lesion when the tooth is dried**

**3: Localized fractures on the enamel surface, no symptoms in dentin**

**4: Yellow-brown reflection of dentin**

**5: Visible presence of cavity enclosing dentin**

**6: Presence of large cavitation covering dentin (more than half of the occlusal surface)**

| **55** | **54** | **53** | **52** | **51** | **61** | **62** | **63** | **64** | **65** |
| --- | --- | --- | --- | --- | --- | --- | --- | --- | --- |
|  |  |  |  |  |  |  |  |  |  |
|  |  |  |  |  |  |  |  |  |  |
| **85** | **84** | **83** | **82** | **81** | **71** | **72** | **73** | **74** | **75** |

**2.3. VISIBLE PLAQUE INDEX**

**THERE IS PLAQUE, THERE IS NO PLAQUE**

**2.4. DDE INDEX**

**1: Opacity: Anomaly in enamel transparency, qualitative defect of enamel**

**2: Hypoplasia: Decrease in enamel thickness, quantitative defect of enamel**

**3: Discolored enamel: The type of discoloration in the enamel**

**4: Developmental defects of enamel: Disturbances in the hard tissue matrix and mineralization throughout odontogenesis**

| **55** | **54** | **53** | **52** | **51** | **61** | **62** | **63** | **64** | **65** |
| --- | --- | --- | --- | --- | --- | --- | --- | --- | --- |
|  |  |  |  |  |  |  |  |  |  |
|  |  |  |  |  |  |  |  |  |  |
| **85** | **84** | **83** | **82** | **81** | **71** | **72** | **73** | **74** | **75** |

**CHAPTER 3 – PATIENT-RELATED ETIOLOGICAL FACTORS**

| **Birth weight** |  | | | | | | | | |  |
| --- | --- | --- | --- | --- | --- | --- | --- | --- | --- | --- |
| **Time of birth** | <37 weeks ( ) | | 37-40 weeks ( ) | | | 40 weeks and more ( ) | | | |  |
| **Birth order** | First child ( ) | | 2.Child ( ) | | | 3 or more ( ) | | | |  |
| **Number of siblings** | 0 ( ) | | 1 ( ) | | | 2 or more ( ) | | | |  |
| **Chronic illness, physical or mental disability?** |  | | | | | | | | |  |
| **Place of residence** | City ( ) | | District ( ) | | | Köy ( ) | | | |  |
| **The person in whom he lives** | Parents ( ) | | | Other ( ) | | | | | |  |
| **Caregiver** | Parents ( ) | | Grandparents( ) | | | Caregiver ( ) | | | |  |
| **Does She Go to Kindergarten or Kindergarten?** | Yes ( ) | | | No ( ) | | | | | |  |
| **Duration of breastfeeding** | Less than 6 months ( ) | | 6-12 months ( ) | | | 12 months and more ( ) | | | |  |
| **Use of Baby Bottles** | Yes ( ) | | | | No ( ) | | | | |  |
| **Adding Sugar to the Bottle?**  **(Honey, molasses, biscuits, etc.)** | Yes ( ) | | | | No ( ) | | | | |  |
| **Breastfeeding** | Less than 6 months ( ) | | 6-12 months ( ) | | | 12 months and more ( ) | | | |  |
| **Transition to Supplementary Food** | 0-6 months ( ) | | 6-12 months ( ) | | | 12. Month and beyond ( ) | | | |  |
| **Dental Cleaning After Night Feeding** | Yes ( ) | | | | No ( ) | | | | |  |
| **Eruption Time of the First Milk Tooth** | Before the 6th month ( ) | | 6.month ( ) | | | After the 6th month ( ) | | | |  |
| **Age to Start Brushing** | 6- 12 months ( ) | | After 1 year of age ( ) | | | After 3 years of age ( ) | | Ever ( ) | |  |
| **Daily Brushing Frequency** | Ever ( ) | | 1 time ( ) | | | 2 or more ( ) | | | |  |
| **Does Your Child Use Toothpaste?** | Yes ( ) | | | | No ( ) | | | | |  |
| **Does the Toothpaste He Uses Contain Fluoride?** | Yes ( ) | | No ( ) | | | I don't know ( ) | | | |  |
| **How is brushing done?** | By parent ( ) | | By the child under parental supervision ( ) | | | Child alone ( ) | | | |  |
| **Have You Ever Had Fluoride Applied?** | Yes ( ) | | No ( ) | | | I don't remember ( ) | | | |  |
| **If Yes, the type of fluorine applied** | Fluorine gel ( ) | | Vernik flower ( ) | | | Flor tablet ( ) | | | |  |
| **How Many Times Has Fluoride Been Applied?** | 1 ( ) | | 2 ( ) | | | 3 or more ( ) | | | |  |
| **Is there an additional preservative you use? (Like GC Tooth Mousse)** | Yes ( ) | | No ( ) | | |  | | | |  |
| **Will Your Child Keep Food in His Mouth?** | Yes ( ) | | | | No ( ) | | | | |  |
| **Does Your Child Chew Gum?** | Yes ( ) | | | | No ( ) | | | | |  |
| **If Yes, Chewing Gum Is Your Preference?** | Sugary ( ) | Sugar Free ( ) | | | | Ksilitollü ( ) | | |  |  |
| **Chewing Gum Time** | Less than 5 minutes ( ) | | | | More than 5 minutes ( ) | | | | |  |
| **Daily Sugary Snack Consumption Frequency** | Ever ( ) | | Less than 3 ( ) | | | 4 or more ( ) | | | |  |
| **Daily Sugary Beverage Consumption Frequency** | Ever ( ) | | Less than 3 ( ) | | | 4 or more ( ) | | | |  |
| **Frequency of Acidic Beverage Consumption** | 1 ( ) per week | | 2 or more per week ( ) | | | Rarely or never ( ) | | | |  |
| **First Dentist Visit** | 0-6 months | | 1-3 years | | | 3 years < | | | |  |
| **Does He Go for Regular Check-ups?** | Yes ( ) | | | | No ( ) | | | | |  |
| **Yes, How Often Does He Go To Work?** | Every 3 months ( ) | Every 6 months ( ) | | | | Once a year ( ) | | |  |  |
| **Has Your Child Received Oral Care Training?** | Yes ( ) | | | | No ( ) | | | | |  |
| **Does he have any bad habits such as thumb sucking, pacifier use, mouth breathing?** | Yes ( ) | | | | No ( ) | | | | |  |
| **Have you dipped the pacifier in honey and sugar and given it?** | Yes ( ) | | | | No ( ) | | | | |  |
| **Has your child had a high fever?** | Yes ( ) | | | | No ( ) | | | | |  |
| **Oral Intake of Antibiotics** | Ever ( ) | 1-3 times ( ) | | | | | 4 and more ( ) | | | |

**CHAPTER 4 – PARENTAL ETIOLOGICAL FACTORS**

| **Mother's Age** |  | | | | | | | | | | | | | |
| --- | --- | --- | --- | --- | --- | --- | --- | --- | --- | --- | --- | --- | --- | --- |
| **Father's Age** |  | | | | | | | | | | | | | |
| **Mother's age at the time of birth** | 18-24 ( ) | | | | | 25-29 ( ) | | | | | | 30 and over ( ) | | |
| **Did the Mother Experience Any Discomfort or Complications During Pregnancy? If yes, what is it?** | Yes ( ) | | | | | | | No ( ) | | | | | | |
| **Have you had febrile illness during pregnancy?** | Yes ( ) | | | | | | | No ( ) | | | | | | |
| **Did you smoke or drink alcohol during pregnancy?** | Yes ( ) | | | | | | | No ( ) | | | | | | |
| **Did You Use Medication During Pregnancy? (other than vitamins)** | Yes ( ) | | | | | | | No ( ) | | | | | | |
| **Mother's Educational Status** | Primary School and Earlier ( ) | Secondary School ( ) | High School ( ) | | | | Associate Degree( ) | | License ( ) | | | | M.Sc. ( ) | Ph.D. ( ) |
| **Father's Educational Status** | Primary School and Earlier ( ) | Secondary School ( ) | High School ( ) | | | | Associate Degree( ) | | License ( ) | | | | M.Sc. ( ) | Ph.D. ( ) |
| **Does mom work?** | Yes ( ) | | | | | | | No ( ) | | | | | | |
| **Does mom brush her teeth?** | Yes ( ) | | | | | | | No ( ) | | | | | | |
| **Mother's Brushing Frequency** | Occasionally ( ) | | | | 1 time per day ( ) | | | | | | 2 or more per day ( ) | | | |
| **Does mom use toothpaste?** | Yes ( ) | | | | | | | No ( ) | | | | | | |
| **Does Mom Floss?** | Yes ( ) | | | | | | | No ( ) | | | | | | |
| **Does dad brush his teeth?** | Yes ( ) | | | | | | | No ( ) | | | | | | |
| **Father's Brushing Frequency** | Occasionally ( ) | | | 1 time per day ( ) | | | | | | 2 or more per day ( ) | | | | |
| **Does dad use toothpaste?** | Yes ( ) | | | | | | | No ( ) | | | | | | |
| **Does Dad Floss?** | Yes ( ) | | | | | | | No ( ) | | | | | | |
| **How Often Do You Visit the Dentist?** | When I experience pain or any distress ( ) | | | Once a year ( ) | | | | | | Every 6 months ( ) | | | | |
| **Toothpaste of choice?** | Fluoride ( ) | | | Fluoride Free ( ) | | | | | | I don't know ( ) | | | | |
| **Have You Received Training on Oral Care?** | Yes ( ) | | | | | | | No ( ) | | | | | | |
| **Do you use the same spoon as your child?** | Yes ( ) | | | | | | | No ( ) | | | | | | |
| **Do you chew your child's food?** | Yes ( ) | | | | | | | No ( ) | | | | | | |
| **Do you think it is necessary to treat milk teeth?** | Yes ( ) | | | No ( ) | | | | | | I have no idea ( ) | | | | |
| **Do you think your child's oral care is sufficient?** | Yes ( ) | | | No ( ) | | | | | | I don't know ( ) | | | | |

1. Hallett KB, O'Rourke PK. Social and behavioural determinants of early childhood caries. Aust Dent J. 2003;48(1):27-33.

2. Jain M, Namdev R, Bodh M, Dutta S, Singhal P, Kumar A. Social and Behavioral Determinants for Early Childhood Caries among Preschool Children in India. J Dent Res Dent Clin Dent Prospects. 2015;9(2):115-20.

3. Li J, Fan W, Zhou Y, Wu L, Liu W, Huang S. The status and associated factors of early childhood caries among 3- to 5-year-old children in Guangdong, Southern China: a provincial cross-sectional survey. BMC Oral Health. 2020;20(1):265.

4. Wulaerhan J, Abudureyimu A, Bao XL, Zhao J. Risk determinants associated with early childhood caries in Uygur children: a preschool-based cross-sectional study. BMC Oral Health. 2014;14:136.

5. Foxman B, Davis E, Neiswanger K, McNeil D, Shaffer J, Marazita ML. Maternal factors and risk of early childhood caries: A prospective cohort study. Community Dent Oral Epidemiol. 2023;51(5):953-65.

6. Kocaman GÜ, Çebi AT. Erken çocukluk çağı çürüklerinin önlenmesinde annelerin oral hijyen alışkanlıkları ve çocuk beslenmesi konusunda bilgi düzeyinin ve farkındalıklarının belirlenmesi. J Süleyman Demirel Üniversitesi Sağlık Bilimleri Dergisi. 2019;10(3):268-72.

7. Boonyawong M, Auychai P, Duangthip D. Risk Factors of Dental Caries in Preschool Children in Thailand: A Cross-Sectional Study. Healthcare (Basel, Switzerland). 2022;10(5).

8. Chouchene F, Masmoudi F, Baaziz A, Maatouk F, Ghedira H. Early Childhood Caries Prevalence and Associated Risk Factors in Monastir, Tunisia: A Cross-Sectional Study. Frontiers in public health. 2022;10:821128.

9. Congiu G, Campus G, Sale S, Spano G, Cagetti MG, Luglie PF. Early childhood caries and associated determinants: a cross-sectional study on Italian preschool children. J Public Health Dent. 2014;74(2):147-52.

10. Ivancevic V, Tusek I, Tusek J, Knezevic M, Elheshk S, Lukovic I. Using association rule mining to identify risk factors for early childhood caries. Comput Methods Programs Biomed. 2015;122(2):175-81.

11. Johansson I, Holgerson PL, Kressin NR, Nunn ME, Tanner AC. Snacking habits and caries in young children. Caries Res. 2010;44(5):421-30.

12. Mattila ML, Rautava P, Sillanpaa M, Paunio P. Caries in five-year-old children and associations with family-related factors. J Dent Res. 2000;79(3):875-81.

13. Narang R, Saha S, G VJ, Kumari M, Mohd S, Saha S. The maternal socioeconomic status and the caries experience among 2-6 years old preschool children of lucknow city, India. Journal of clinical and diagnostic research : JCDR. 2013;7(7):1511-3.

14. Onur SG, Kargul B. Assessment of potential risk factors associated with early childhood caries in a subpopulation of children from Thrace region of Turkey. Folia Med (Plovdiv). 2021;63(4):546-56.

15. Pinto GDS, Azevedo MS, Goettems ML, Correa MB, Pinheiro RT, Demarco FF. Are Maternal Factors Predictors for Early Childhood Caries? Results from a Cohort in Southern Brazil. Braz Dent J. 2017;28(3):391-7.

16. Sujlana A, Pannu PK. Family related factors associated with caries prevalence in the primary dentition of five-year-old children. J Indian Soc Pedod Prev Dent. 2015;33(2):83-7.

17. Tanaka K, Miyake Y, Sasaki S. The effect of maternal smoking during pregnancy and postnatal household smoking on dental caries in young children. J Pediatr. 2009;155(3):410-5.

18. Tinanoff N, Baez RJ, Diaz Guillory C, Donly KJ, Feldens CA, McGrath C, et al. Early childhood caries epidemiology, aetiology, risk assessment, societal burden, management, education, and policy: Global perspective. Int J Paediatr Dent. 2019;29(3):238-48.

19. Upadhyay S, Dahal S. Assessing the Relationship of Maternal Factors and Family Income with Early Childhood Caries: A Hospital Based Study. Kathmandu Univ Med J (KUMJ). 2017;15(60):288-91.

20. Yiğit T. The effects of parent’s oral hygiene habits and socio-economic status on early childhoos caries Atatürk Üniversitesi Diş Hekimliği Fakültesi Dergisi 2020;30 (3):366-72.

21. dos Santos Junior VE, de Sousa RMB, Oliveira MC, de Caldas Junior AF, Rosenblatt A. Early childhood caries and its relationship with perinatal, socioeconomic and nutritional risks: a cross-sectional study. BMC Oral Health. 2014;14:1-5.
