# Supplementary material for: The effects of familial factors on the early childhood caries of preschool children: a cross-sectional study
Source: BMC Oral Health. 2025 Jun 5;25:920. doi: 10.1186/s12903-025-06140-w (PMC12142995; doi:10.1186/s12903-025-06140-w)
Supplement: Supplementary file 2 — Supplementary Material 2 [file 12903_2025_6140_MOESM2_ESM.pdf]

# Certificate of Proofreading

**This document certifies that the manuscript detailed below was edited and proofread for correct English grammar, spelling, punctuation, and vocabulary at the Proofreading & Editing Office.**

"The effects of familial factors on the early childhood caries of preschool children: a cross-sectional study"

**By**

Zeynep Aslı Güçlü, Cansu Bilge Karadağ

**December 2, 2024**

English Editor
Prof. Dr. Eugene Steele

**Proofreading & Editing Office**
**Office of the Dean for Research**
**Erciyes University**

English Editor
Lecturer Aysegul Ozaslan, M.A.

**Certificate Number:** 2024.246 **E-mail:** editingoffice@erciyes.edu.tr **Contact:** +90-352-207-66-66 (Ext: 12508)
