# Supplementary material for: The effects of familial factors on the early childhood caries of preschool children: a cross-sectional study
Source: BMC Oral Health. 2025 Jun 5;25:920. doi: 10.1186/s12903-025-06140-w (PMC12142995; doi:10.1186/s12903-025-06140-w)
Supplement: Supplementary file 3 — Supplementary Material 3 [file 12903_2025_6140_MOESM3_ESM.docx]

**Tables**

**Table 1:** Findings related to children

|  | **Frequency (n)/Mean ± std. deviation** | **Percentage (%)/median (min. - max.)** |
| --- | --- | --- |
| **Age** |  |  |
| **13-24 Months(1 Years old)** | 4 | 1 |
| **25-36 Months(2 Years old)** | 17 | 4. 2 |
| **37-48 Months(3 Years old)** | 73 | 18 |
| **49-60 Months(4 Years old)** | 124 | 30. 6 |
| **61-72 Months(5 Years old)** | 187 | 46. 2 |
| **Age (month)** | 56. 75 ± 11. 24 | 59. 00 (13. 00 - 71. 00) |
| **Gender** |  |  |
| **Boy** | 191 | 47. 2 |
| **Girl** | 214 | 52. 8 |
| **DMFT** | 8. 79 ± 4. 33 | 9. 00 (0. 00 - 20. 00) |
| **ICDAS** | 5. 06 ± 1. 46 | 5. 00 (0. 00 - 6. 00) |
| **HAS ECC** |  |  |
| **Yes** | 387 | 95. 6 |
| **No** | 18 | 4. 4 |

|  | **Frequency (n)** | **Percentage (%)** |
| --- | --- | --- |
| **Dental Cleaning After Night Feeding** |  |  |
| **Yes** | 149 | 36. 8 |
| **No.** | 256 | 63. 2 |
| **Age of Starting Brushing** |  |  |
| **6- 12 Months** | 13 | 3. 2 |
| **After 1 year of age** | 171 | 42. 2 |
| **After 3 years of age** | 172 | 42. 5 |
| **Never** | 49 | 12. 1 |
| **Daily Brushing Frequency** |  |  |
| **Never** | 49 | 12. 13 |
| **1 Time** | 300 | 74. 07 |
| **2 or More** | 56 | 13. 8 |
| **When is Brushing?** |  |  |
| **Morning** | 74 | 20. 8 |
| **Evening** | 140 | 39. 3 |
| **Changes** | 86 | 24. 1 |
| **Morning and Evening** | 56 | 15. 7 |
| **Does he/she use toothpaste for every brushing?** |  |  |
| **Yes** | 331 | 93 |
| **No.** | 25 | 7 |
| **Does Toothpaste Contain Fluorine?** |  |  |
| **Yes.** | 67 | 18. 8 |
| **No.** | 112 | 31. 7 |
| **I do not know** | 177 | 49. 5 |
| **How is brushing done?** |  |  |
| **By Parent** | 67 | 18. 8 |
| **By Child under Parental Supervision** | 208 | 58. 4 |
| **Child Alone** | 81 | 22. 8 |

**Table 2:** Frequency distribution of factors related to the mother

|  | **FREQUENCY (n)** | **Percentage (%)** |
| --- | --- | --- |
| **Age of the mother at the time of birth** |  |  |
| **18-24** | 106 | 26,2 |
| **25-29** | 167 | 41,2 |
| **30 and Above** | 132 | 32,6 |
| **Mother's Education Level** |  |  |
| **Primary School and Less** | 63 | 15,6 |
| **Middle School** | 96 | 23,7 |
| **High School** | 118 | 29,1 |
| **Associate Degree** | 51 | 12,6 |
| **Bachelor's degree** | 65 | 16 |
| **Master's Degree** | 9 | 2,2 |
| **PhD** | 3 | 0,7 |
| **Does the mother work?** |  |  |
| **Yes** | 73 | 18 |
| **No** | 332 | 82 |
| **Does the Mother Brush Her Teeth** |  |  |
| **Yes** | 385 | 95,1 |
| **No** | 20 | 4,9 |
| **Mother's Brushing Frequency** |  |  |
| **Occasionally** | 44 | 11,4 |
| **Once a day** | 212 | 55,06 |
| **2 or More Per Day** | 129 | 33,54 |
| **Does the Mother Use Toothpaste** |  |  |
| **Yes** | 382 | 99,2 |
| **No** | 3 | 0,8 |
| **Does the Mother Use Dental Floss** |  |  |
| **Yes** | 84 | 20,7 |
| **No** | 321 | 79,3 |

**Table 3:** Frequency distribution of father-related factors

|  | **FREQUENCY (n)** | **Percentage (%)** |
| --- | --- | --- |
| **Father's Education Level** |  |  |
| **Primary School and Before** | 53 | 13,3 |
| **Middle School** | 68 | 17 |
| **High School** | 141 | 35,3 |
| **Associate Degree** | 33 | 8,3 |
| **Bachelor's degree** | 81 | 20,3 |
| **Master's Degree** | 17 | 4,3 |
| **PhD** | 6 | 1,5 |
| **Does the Father Brush His Teeth** |  |  |
| **Yes** | 351 | 88 |
| **No.** | 48 | 12 |
| **Father's Brushing Frequency** |  |  |
| **Occasionally** | 85 | 24,2 |
| **Once a day** | 175 | 49,8 |
| **2 or More Per Day** | 91 | 26 |
| **Does the Father Use Toothpaste** |  |  |
| **Yes** | 339 | 96,4 |
| **No.** | 12 | 3,6 |
| **Does the Father Use Dental Floss** |  |  |
| **Yes** | 82 | 20,6 |
| **No.** | 317 | 79,4 |

**Table 4:** Comparison of DMFT and ICDAS scores according to maternal factors

|  | | **DMFT** | | | | **ICDAS** | | |
| --- | --- | --- | --- | --- | --- | --- | --- | --- |
|  | Mean ± Std. deviation | | Median (min. - max.) | | Mean ± Std. deviation | | Median (min. - max.) | |
| **Mother's Education Level** |  | |  | |  | |  | |
| **Primary school and before** | 9,95 ± 3,62a | | 10,00 (2,00 - 20,00) | | 5,56 ± 0,64 | | 6,00 (4,00 - 6,00)b | |
| **Middle School** | 9,67 ± 3,31a | | 10,00 (2,00 - 19,00) | | 5,50 ± 0,77 | | 6,00 (2,00 - 6,00)b | |
| **High School** | 8,78 ± 4,50ab | | 9,00 (0,00 - 20,00) | | 5,03 ± 1,46 | | 5,00 (0,00 - 6,00)ab | |
| **Associate degree** | 8,41 ± 4,61ab | | 8,00 (0,00 - 20,00) | | 4,86 ± 1,55 | | 5,00 (0,00 - 6,00)ab | |
| **Bachelor's degree and above** | 7,01 ± 4,98b | | 7,00 (0,00 - 20,00) | | 4,27 ± 2,06 | | 5,00 (0,00 - 6,00)a | |
| **Test stat.** | | 5,321 | | | | 31,635 | | |
| **p**** | | **<0,001** | | | | **<0,001** | | |
| **Does the mother work?** | |  | |  | |  | |  |
| **Yes** | | 6,45 ± 4,71 | | 6,00 (0,00 - 16,00) | | 4,18 ± 2,15 | | 5,00 (0,00 - 6,00) |
| **No.** | | 9,30 ± 4,08 | | 9,00 (0,00 - 20,00) | | 5,25 ± 1,17 | | 6,00 (0,00 - 6,00) |
| **Test stat.** | | 16285,000 | | | | 15735,500 | | |
| **p*** | | **<0,001** | | | | **<0,001** | | |
| **Does the Mother Use Dental Floss?** | |  | |  | |  | |  |
| **Yes** | | 7,51 ± 4,56 | | 8,00 (0,00 - 20,00) | | 4,57 ± 1,66 | | 5,00 (0,00 - 6,00) |
| **No.** | | 9,12 ± 4,22 | | 9,00 (0,00 - 20,00) | | 5,19 ± 1,37 | | 6,00 (0,00 - 6,00) |
| **Test stat.** | | 16342,500 | | | | 17447,000 | | |
| **p*** | | **0,003** | | | | **<0,001** | | |

*Mann‒Whitney U test, **One-way analysis of variance, a--b: There is no difference between groups with the same letter, ---: Not compared due to insufficient observations.

**Table 5:** Comparison of DMFT and ICDAS scores according to father-related factors

|  | | **DMFT** | | | **ICDAS** | |
| --- | --- | --- | --- | --- | --- | --- |
|  | Mean ± Std. deviation | | Median (min. - max.) | Mean ± Std. deviation | | Median (min. - max.) |
| **Father's Education Level** |  | |  |  | |  |
| **Primary school and less** | 10,34 ± 3,18 | | 10,00 (6,00 - 20,00)a | 5,57 ± 0,69 | | 6,00 (3,00 - 6,00)a |
| **Middle School** | 9,46 ± 4,23 | | 9,00 (0,00 - 20,00)a | 5,46 ± 0,95 | | 6,00 (0,00 - 6,00)a |
| **High School** | 8,99 ± 4,19 | | 9,00 (0,00 - 20,00)a | 5,27 ± 1,16 | | 6,00 (0,00 - 6,00)a |
| **Associate degree** | 9,33 ± 4,48 | | 10,00 (2,00 - 18,00)ab | 5,12 ± 1,14 | | 5,00 (1,00 - 6,00)ab |
| **Bachelor's degree and above** | 6,91 ± 4,55 | | 7,00 (0,00 - 16,00)b | 4,19 ± 2,04 | | 5,00 (0,00 - 6,00)b |
| **Test stat.** | | 23,296 | | | 42,165 | |
| **p**** | | **<0,001** | | | **<0,001** | |
| **Does the Father Brush His Teeth** |  | |  |  | |  |
| **Yes** | 8,52 ± 4,34 | | 8,00 (0,00 - 20,00) | 4,97 ± 1,51 | | 5,00 (0,00 - 6,00) |
| **No.** | 10,29 ± 4,04 | | 11,00 (0,00 - 19,00) | 5,58 ± 0,96 | | 6,00 (0,00 - 6,00) |
| **Test stat.** | | 10507,000 | | | 10846,000 | |
| **p*** | | **0,005** | | | **<0,001** | |
| **Father's Brushing Frequency** |  | |  |  | |  |
| **Occasionally** | 9,41 ± 4,19 | | 9,00 (0,00 - 20,00) | 5,19 ± 1,21 | | 5,00 (0,00 - 6,00)b |
| **1 time a day** | 8,73 ± 4,30 | | 9,00 (0,00 - 20,00) | 5,04 ± 1,52 | | 5,00 (0,00 - 6,00)b |
| **2 or more per day** | 7,74 ± 4,67 | | 8,00 (0,00 - 20,00) | 4,66 ± 1,70 | | 5,00 (0,00 - 6,00)a |
| **Test stat.** | | 5,659 | | | 7,216 | |
| **p**** | | 0,059 | | | **0,027** | |
| **Does the Father Use Dental Floss** | Does the Father Use Dental Floss | |  |  | |  |
| **Yes** | Yes | | 8,00 (0,00 - 19,00) | 4,52 ± 1,77 | | 5,00 (0,00 - 6,00) |
| **No.** | No. | | 9,00 (0,00 - 20,00) | 5,18 ± 1,35 | | 6,00 (0,00 - 6,00) |
| **Test stat.** | | Test stat. | | | 16297,000 | |
| **p*** | | **0,009** | | | **<0,001** | |

*Mann‒Whitney U test, **Kruskal‒Wallis test, a-b: There is no difference between groups with the same letter.

**Table 6:** Comparison results according to mothers’ education level

|  | **Primary school and belove** | **Middle School** | **High School** | **Associate degree** | **Bachelor's degree and above** | **Test stat.** | **p** |
| --- | --- | --- | --- | --- | --- | --- | --- |
| **Brushing Frequency of Children** |  |  |  |  |  |  |  |
| **Never.** | 9 (14,3) | 17 (17,7) | 19 (16,1) | 4 (7,8) | 5 (6,5) | 8,75 | 0,36 |
| **Once** | 47 (74,6) | 65 (67,7) | 84 (71,2) | 37 (72,5) | 62 (80,5) |  |  |
| **2 or more** | 7 (11,1) | 14 (14,6) | 15 (12,7) | 10 (19,6) | 10 (13) |  |  |
| **Frequency of Parental Visits to the Dentist** |  |  |  |  |  |  |  |
| **When I experience pain or any kind of distress** | 49 (77,8) | 80 (83,3) | 101 (85,6) | 39 (76,5) | 53 (68,8) | 11,48 | 0,18 |
| **Once a year** | 13 (20,6) | 12 (12,5) | 14 (11,9) | 9 (17,6) | 20 (26) |  |  |
| **Every 6 months** | 1 (1,6) | 4 (4,2) | 3 (2,5) | 3 (5,9) | 4 (5,2) |  |  |
| **Parents' Choice of Toothpaste** |  |  |  |  |  |  |  |
| **Fluoride** | 8 (12,7)a | 24 (25)a | 23 (19,5)a | 15 (29,4)ab | 36 (46,8)b | 37,59 | **<0,01** |
| **Fluoride Free** | 8 (12,7) | 10 (10,4) | 19 (16,1) | 9 (17,6) | 17 (22,1) |  |  |
| **I do not know** | 47 (74,6)a | 62 (64,6)a | 76 (64,4)a | 27 (52,9)ab | 24 (31,2)b |  |  |
| **Do You Use the Same Spoon with Your Child?** |  |  |  |  |  |  |  |
| **Yes** | 6 (9,5) | 16 (16,7) | 14 (11,9) | 4 (7,8) | 14 (18,2) | 4,83 | 0,31 |
| **No.** | 57 (90,5) | 80 (83,3) | 104 (88,1) | 47 (92,2) | 63 (81,8) |  |  |
| **Do you chew your child's food?** |  |  |  |  |  |  |  |
| **Yes.** | 2 (3,2) | 5 (5,2) | 9 (7,7) | 1 (2) | 2 (2,6) | 4,33 | 0,36 |
| **No.** | 61 (96,8) | 91 (94,8) | 108 (92,3) | 50 (98) | 75 (97,4) |  |  |
| **Do you think it is necessary to treat deciduous teeth?** |  |  |  |  |  |  |  |
| **Yes.** | 32 (50,8) | 61 (63,5) | 72 (61) | 33 (64,7) | 61 (79,2) | 15,34 | 0,05 |
| **No.** | 6 (9,5) | 5 (5,2) | 6 (5,1) | 5 (9,8) | 3 (3,9) |  |  |
| **No opinion** | 25 (39,7) | 30 (31,3) | 40 (33,9) | 13 (25,5) | 13 (16,9) |  |  |
| **Do you think your child's oral care is adequate?** |  |  |  |  |  |  |  |
| **Yes** | 5 (7,9) | 12 (12,5) | 16 (13,6) | 11 (21,6) | 14 (18,2) | 15,38 | 0,05 |
| **No.** | 42 (66,7) | 60 (62,5) | 70 (59,3) | 35 (68,6) | 54 (70,1) |  |  |
| **I do not know** | 16 (25,4) | 24 (25) | 32 (27,1) | 5 (9,8) | 9 (11,7) |  |  |

*Chi-square test, a-b: No difference between groups with the same letter, frequency (percentage)

**Table 7:** Comparison results according to fathers’ education level

|  | **Primary school and belove** | **Middle School** | **High School** | **Associate degree** | **Bachelor's degree and above** | **Test stat.** | **p** |
| --- | --- | --- | --- | --- | --- | --- | --- |
| **Brushing Frequency of Children** |  |  |  |  |  |  |  |
| **Never.** | 11 (20,8) | 11 (16,2) | 21 (14,9) | 2 (6,1) | 8 (7,7) | 11,63 | 0,17 |
| **Once** | 33 (62,3) | 44 (64,7) | 103 (73) | 26 (78,8) | 84 (80,8) |  |  |
| **2 or more** | 9 (17) | 13 (19,1) | 17 (12,1) | 5 (15,2) | 12 (11,5) |  |  |
| **Frequency of Parental Visits to the Dentist** |  |  |  |  |  |  |  |
| **When I experience pain or any kind of distress** | 43 (81,1) | 56 (82,4) | 114 (80,9) | 25 (75,8) | 80 (76,9) | 4,33 | 0,83 |
| **Once a year** | 9 (17) | 9 (13,2) | 22 (15,6) | 8 (24,2) | 19 (18,3) |  |  |
| **Every 6 months** | 1 (1,9) | 3 (4,4) | 5 (3,5) | 0 (0) | 5 (4,8) |  |  |
| **Parents' Choice of Toothpaste** |  |  |  |  |  |  |  |
| **Fluoride** | 7 (13,2)a | 12 (17,6)a | 35 (24,8)ab | 10 (30,3)ab | 41 (39,4)b | 28,51 | **<0,01** |
| **Fluoride Free** | 6 (11,3) | 8 (11,8) | 21 (14,9) | 4 (12,1) | 23 (22,1) |  |  |
| **I do not know** | 40 (75,5)a | 48 (70,6)a | 85 (60,3)a | 19 (57,6)ab | 40 (38,5)b |  |  |
| **Do You Use the Same Spoon with Your Child?** |  |  |  |  |  |  |  |
| **Yes** | 8 (15,1) | 13 (19,1) | 20 (14,2) | 0 (0) | 11 (10,6) | 8,08 | 0,09 |
| **No.** | 45 (84,9) | 55 (80,9) | 121 (85,8) | 33 (100) | 93 (89,4) |  |  |
| **Do you chew your child's food?** |  |  |  |  |  |  |  |
| **Yes.** | 2 (3,8) | 2 (2,9) | 9 (6,4) | 1 (3) | 4 (3,8) | 1,92 | 0,75 |
| **No.** | 51 (96,2) | 66 (97,1) | 131 (93,6) | 32 (97) | 100 (96,2) |  |  |
| **Do you think it is necessary to treat deciduous teeth?** |  |  |  |  |  |  |  |
| **Yes.** | 33 (62,3) | 40 (58,8) | 78 (55,3) | 26 (78,8) | 76 (73,1) | 12,85 | 0,12 |
| **No.** | 4 (7,5) | 4 (5,9) | 10 (7,1) | 1 (3) | 6 (5,8) |  |  |
| **No opinion** | 16 (30,2) | 24 (35,3) | 53 (37,6) | 6 (18,2) | 22 (21,2) |  |  |
| **Do you think your child's oral care is adequate?** |  |  |  |  |  |  |  |
| **Yes** | 6 (11,3) | 8 (11,8) | 14 (9,9) | 6 (18,2) | 23 (22,1) | 12,08 | 0,15 |
| **No.** | 38 (71,7) | 44 (64,7) | 90 (63,8) | 19 (57,6) | 65 (62,5) |  |  |
| **I do not know** | 9 (17) | 16 (23,5) | 37 (26,2) | 8 (24,2) | 16 (15,4) |  |  |

*Chi-square test, a-b: No difference between groups with the same letter, frequency (percentage)

**Table 8**. Investigation of the effect of independent variables on DMFT by Robust regression analysis

|  | β1 (95% CI) | Q. Error | β2 | t | p | VIF |
| --- | --- | --- | --- | --- | --- | --- |
| Constant | 7 (4,83 - 9,16) | 1,10 | 0,00 | 6,36 | **<0.01** | --- |
| Mother's age at the time of birth (ref: 18-24) |  |  |  |  |  |  |
| 25-29 | -0,76 (-1,75 - 0,23) | 0,50 | -0,09 | -1,51 | 0,13 | 1,69 |
| 30 and above | -0,49 (-1,6 - 0,63) | 0,57 | -0,06 | -0,86 | 0,39 | 1,95 |
| Mother's Educational Status (Reference: Primary school and before) |  |  |  |  |  |  |
| Secondary school | 0,28 (-1,13 - 1,68) | 0,72 | 0,03 | 0,38 | 0,70 | 2,64 |
| High school | -0,57 (-1,95 - 0,81) | 0,70 | -0,06 | -0,81 | 0,42 | 2,73 |
| Associate Degree | -0,3 (-1,96 - 1,37) | 0,85 | -0,02 | -0,35 | 0,73 | 2,20 |
| Bachelor's degree and above | 0,27 (-1,53 - 2,07) | 0,92 | 0,03 | 0,29 | 0,77 | 3,55 |
| Does the mother work (reference: yes) | 2,37 (1,15 - 3,59) | 0,62 | 0,22 | 3,83 | **<0.01** | 1,54 |
| Does Mom Floss (Reference: Yes) | 0,67 (-0,38 - 1,73) | 0,54 | 0,07 | 1,25 | 0,21 | 1,29 |
| Father's Educational Status (Reference: Primary school and before) |  |  |  |  |  |  |
| Secondary school | -0,91 (-2,4 - 0,58) | 0,76 | -0,08 | -1,20 | 0,23 | 2,24 |
| High school | -0,97 (-2,28 - 0,34) | 0,67 | -0,11 | -1,46 | 0,15 | 2,78 |
| Associate Degree | -0,21 (-2,04 - 1,61) | 0,93 | -0,01 | -0,23 | 0,82 | 1,81 |
| Bachelor's degree and above | -1,83 (-3,44 - -0,22) | 0,82 | -0,20 | -2,23 | **0,03** | 3,56 |
| Does dad brush his teeth (reference: yes) | 1,24 (0,06 - 2,43) | 0,60 | 0,10 | 2,06 | **0,04** | 1,07 |
| Does Dad Floss (Reference: Yes) | 0,69 (-0,36 - 1,74) | 0,53 | 0,07 | 1,29 | 0,20 | 1,28 |

F=4.91, p<0.01, R2=0.152, β1 (95% CI): Non-standardized beta coefficient (95% confidence interval), β2: Standardized beta coefficient

The regression model established to examine the effect of independent variables on DMFT values was statistically significant (F=4.91, p<0.01). DMFT values of non-working mothers were obtained 2.37 units higher than those who worked (p<0.01). DMFT values of undergraduate and above were 1.83 units less than those in primary school and before (p=0.03). DMFT values of fathers who did not brush their teeth were 1.24 units higher than those who brushed (p=0.04). Other variables did not have a statistically significant effect on DMFT values (p>0.05). Together with the independent variables in the model, the dependent variable is explained at a rate of 15.2%.

**Table 9.** Robust regression analysis of the effect of independent variables on ICDAS

|  | β1 (95% CI) | Q. Error | β2 | t | p | VIF |
| --- | --- | --- | --- | --- | --- | --- |
| Constant | 4,85 (4,36 - 5,35) | 0,25 | 0,00 | 19,31 | **<0.01** |  |
| Mother's age at the time of birth (ref: 18-24) |  |  |  |  |  |  |
| 25-29 | -0,02 (-0,24 - 0,2) | 0,11 | -0,01 | -0,17 | 0,87 | 1,64 |
| 30 and above | 0 (-0,25 - 0,24) | 0,13 | 0,00 | -0,03 | 0,98 | 1,93 |
| Mother's Educational Status (Reference: Primary school and before) |  |  |  |  |  |  |
| Secondary school | 0,05 (-0,26 - 0,36) | 0,16 | 0,02 | 0,32 | 0,75 | 2,62 |
| High school | -0,14 (-0,45 - 0,16) | 0,15 | -0,07 | -0,94 | 0,35 | 2,65 |
| Associate Degree | -0,15 (-0,51 - 0,21) | 0,18 | -0,06 | -0,83 | 0,41 | 2,10 |
| Bachelor's degree and above | 0,04 (-0,36 - 0,43) | 0,20 | 0,01 | 0,17 | 0,86 | 3,17 |
| Does the mother work (reference: yes) | 0,32 (0,04 - 0,59) | 0,14 | 0,13 | 2,29 | **0,02** | 1,40 |
| Does Mom Floss (Reference: Yes) | 0,33 (0,09 - 0,56) | 0,12 | 0,14 | 2,71 | **0,01** | 1,25 |
| Father's Educational Status (Reference: Primary school and before) |  |  |  |  |  |  |
| Secondary school | -0,04 (-0,36 - 0,28) | 0,16 | -0,02 | -0,24 | 0,81 | 2,25 |
| High school | -0,08 (-0,36 - 0,21) | 0,15 | -0,04 | -0,52 | 0,60 | 2,75 |
| Associate Degree | -0,07 (-0,47 - 0,33) | 0,20 | -0,02 | -0,34 | 0,73 | 1,78 |
| Bachelor's degree and above | -0,5 (-0,86 - -0,15) | 0,18 | -0,23 | -2,77 | **0,01** | 3,21 |
| Does dad brush his teeth (reference: yes) | 0,24 (-0,02 - 0,49) | 0,13 | 0,09 | 1,83 | 0,07 | 1,07 |
| Does Dad Floss (Reference: Yes) | 0,18 (-0,06 - 0,42) | 0,12 | 0,08 | 1,48 | 0,14 | 1,26 |

F=5.05, p<0.01, R2=0.156, β1 (95% CI): Non-standardized beta coefficient (95% confidence interval), β2: Standardized beta coefficient

The regression model established to examine the effect of independent variables on ICDAS values was statistically significant (F=5.05, p<0.01). ICDAS values of non-working mothers were obtained 0.32 units higher than those who worked (p=0.02). The ICDAS values of mothers who did not use dental floss were 0.33 units higher than those who used dental floss (p=0.01). ICDAS values of undergraduate and above were 0.5 units less than those in primary school and before (p=0.01). There was no statistically significant effect of other variables on ICDAS values (p>0.050). Together with the independent variables in the model, the dependent variable is explained at a rate of 15.6%.

A statistically significant difference was found between the mean DMFT scores according to the educational status of the mothers (p<0.001). The mean DMFT scores of children whose mothers graduated from primary school and before (9.95 ± 3.62) and secondary school (9.67 ± 3.31) were found to be significantly higher than the mean DMFT scores of children whose mothers graduated from undergraduate school and above (7.01 ± 4.98). A statistically significant difference was found between the mean ICDAS scores according to the mother’s educational status (p<0.001). The mean ICDAS score (5.56 ± 0.64) of children whose mothers graduated from primary school and before and the mean ICDAS score (5.50 ± 0.77) of children whose mothers graduated from secondary school were significantly higher than the mean ICDAS score (4.27 ± 2.06) of children whose mothers graduated from undergraduate school and above. The mean DMFT and ICDAS scores decreased as the level of education increased (Table 4). A statistically significant difference was found between the mean DMFT score (p<0.001) and the mean ICDAS score (p<0.001) according to mothers’ employment status. The mean DMFT (6.45 ± 4.71) and ICDAS (4.18 ± 2.15) scores of the children of working mothers were significantly lower than the mean DMFT (9.30 ± 4.08) and ICDAS (5.27 ± 1.17) scores of the children of nonworking mothers (Table 4).

Statistics:

In the study, whether the dependent variables, ICDAS and DMFT scores, showed a significant difference according to different categorical variables was primarily analyzed using comparison tests. As a result of these comparisons, the variables that showed a statistically significant difference were included as independent variables in the multiple regression models. The selection of independent variables in the regression models was based on these preliminary analyses. Additionally, whether there is a multicollinearity problem among the independent variables in the multiple regression models has been evaluated using Variance Inflation Factor (VIF) values. As a result of this analysis, it was determined that there is a correlation within acceptable limits among the independent variables and that there is no multicollinearity problem. In the regression analysis, ICDAS and DMFT values were used as dependent variables. The aim of the study is to examine the effect of independent variables on these values. However, since the dependent variables do not show a normal distribution, robust regression analysis has been preferred to avoid violating the assumptions of classical regression methods. Robust regression provides more reliable results in cases where the data do not meet the normality assumption. The 'VIF' (Variance Inflation Factor) values in Tables 8 and 9 have been included to demonstrate no multicollinearity problem in the analysis models. VIF is a measure that checks whether there is a high correlation among independent variables and is an essential metric for evaluating the model's validity. As a result of the analysis, all VIF values were found to be below acceptable limits, confirming no multicollinearity problem in the model. In this way, the model's reliability and the results' validity have been enhanced.
